# Cultural adaptation and validation of the quality of life questionnaire for patients with actinic keratosis (AKQoL-BR) to Brazilian Portuguese^☆^

**DOI:** 10.1016/j.abd.2021.08.011

**Published:** 2022-09-20

**Authors:** Marcella Akemi Haruno de Vilhena, Ivanka Miranda de Castro, Anna Carolina Miola, Ingrid Stresser Gioppo, Amanda Soares Teixeira, Hélio Amante Miot

**Affiliations:** a Private Practice, São Paulo, SP, Brazil; b Department of Dermatology, Faculty of Medicine, Universidade Estadual Paulista, Botucatu, SP, Brazil; c Hospital Geral de Curitiba, Curitiba, PR, Brazil; d Department of Dermatology, Instituto Lauro de Souza Lima, Bauru, SP, Brazil

Dear Editor,

Actinic keratoses (AK) account for 10% of complaints at dermatological consultations in Brazil.^1^ Although the annual risk of progression from AK to squamous cell carcinoma (SCC) is 0.025%‒0.6%,^2^ patients with multiple AK have up to a 20% risk for the emergence of SCCs.^3^ Moreover, AK can impact the Quality of Life (QoL) of the affected patients.

AK affects photoexposed areas and can cause pain, bleeding, and progress to SCC. To measure the impact of AK on patient QoL, a specific instrument called the Actinic Keratosis Quality of Life questionnaire (AKQoL) was developed in Denmark.^4^ It is a questionnaire with nine self-administered questions, focused on the discomfort caused by AK in the previous week, which has been adapted to other languages.^5^ To date, there is no version adapted for the Portuguese language.

This study aimed to validate and culturally adapt the AKQoL to Brazilian Portuguese. For this purpose, a methodological study was carried out between March 2020 and June 2021, involving outpatients of the Dermatology services of the Faculty of Medicine of Botucatu - UNESP and Instituto Lauro de Souza Lima - ILSL, who were over 18 years old and literate. The project was approved by the Ethics Committee of the institutions.

After the authors' consent, the AKQoL was translated into Portuguese by three dermatologists fluent in English, as recommended by the ISPOR task force.^6^ The translations were merged into a consensual English version aiming to assess the maintenance of its meaning and, subsequently, submitted to cultural adaptation based on the interview of 15 participants with AK, aiming the selection of the most appropriate terms for the understanding of each item.

The translated and culturally adapted version (AKQoL-BR) was applied to 113 participants with AK, sampled by convenience, for psychometric validation. Fifteen of these participants were resubmitted to the questionnaire within seven days to assess its temporal stability (test-retest). Another ten were treated with liquid nitrogen and reassessed after 30 days to assess sensitivity to change. The sample size calculation was based on the COSMIN (Consensus-based Standards for the selection of health Measurement Instruments Study Design Checklist), which recommends the minimum number of 100 assessed subjects for questionnaire validation studies.^7^

The internal consistency of the AKQoL-BR was evaluated using the McDonald-ω coefficient (adequate if ω > 0.70). Its dimensionality was estimated using Horn parallel analysis and confirmed by the UniCo (unidimensional congruence) indicator, the unidimensionality of wich is defined by values ​​≥ 0.95. Temporal stability was tested by the Intraclass Correlation Coefficient (ICC; adequate if ICC > 0.70), and post-treatment responsiveness was assessed by the Wilcoxon test.^8^ The correlation between AK severity and the score was assessed by Spearman rho coefficient.^9^ A p-value ≤ 0.05 was considered significant.

The participants main demographic data are shown in Table 1. The high prevalence of elderly individuals, low phototypes, and high frequency of skin cancer are highlighted. The AKQoL-BR (Table 2) showed good understanding by the participants, and all the questionnaires were completed in less than ten minutes.

**Table 1:** Demographic data of the interviewed patients (n = 113).

Age (years)*		72 (10)
Sex	Female	48 (43%)
	Male	63 (57%)
Phototype	I	16 (14%)
	II	70 (63%)
	III	21 (19%)
	IV	4 (4%)
Level of schooling	Elementary school	71 (69%)
	High School	27 (24%)
	Higher Education	8 (7%)
Smoking		19 (17%)
Sunscreen use		81 (73%)
History of skin tumors		80 (72%)
Previous sun exposure	Mild	15 (14%)
	Moderate	28 (25%)
	Intense	68 (61%)

* Mean(dp)

**Table 2:** Quality of life questionnaire for patients with actinic keratoses – AKQoL-BR.

	**These questions focus on how sun-damaged skin may have affected your life over the past week:**			
	**Please, mark the alternative you consider the correct answer with an X**			
In the past week, having to protect my skin every time I go out in the sun has bothered me.	Not at all	A little	Quite a lot	Very much
In the past week, having sun-damaged skin has made me consider the important things in life.	Not at all	A little	Quite a lot	Very much
In the past week, my quality of life has decreased due to the sun damage to my skin.	Not at all	A little	Quite a lot	Very much
In the past week, I have been worried that the sun damage to my skin will become a more serious skin disease.	Not at all	A little	Quite a lot	Very much
In the past week, I have tried to hide my sun-damaged skin from other people by using make-up or clothes to cover it.	Rarely/ Not at all	Sometimes	Frequently	Constantly
In the past week, I have felt guilty about the sun damage to my skin.	Rarely/ Not at all	Sometimes	Frequently	Constantly
In the past week, I have checked my skin and looked for lesions caused by the sun.	Rarely/ Not at all	Sometimes	Frequently	Very often
In the past week, my life has become more difficult due to the sun damage to my skin.	Not at all	A little	Quite a lot	Very much
In the past week, I have thought about what I have to do when I am exposed to the sun.	Rarely/ Not at all	Sometimes	Frequently	Constantly

The internal consistency of the questionnaire was 0.82 (95%CI 0.78‒0.87), and its unidimensionality was indicated by Horn analysis, with the first factor being responsible for 57.5% of the construct variance, and item 4 (fear of evolution to something serious) was the one with the highest factor loading (0.82). The Kaiser-Meyer-Olkin test resulted in 0.82 and Bartlett statistic was 469 (p < 0.01). The UniCo indicator resulted in 0.955.

There was a good correlation between the items and the total score (rho ≥ 0.45); however, some weak (rho < 0.30) inter-item correlations were observed (Table 3). Item 5, related to the camouflage of AK, was the only one that showed a floor effect, that is, the vast majority of respondents marked the smallest possible measure as an answer to the question asked (in this case, “during the last week, I tried to hide the sun damage to my skin from other people with makeup or clothes”), with 73% of options in the alternative “never or not at all”.

**Table 3:** Inter-item correlation coefficient and Spearman rho correlation coefficient between the items and the total AKQoL-BR score (n = 113).

Variable	Q1	Q2	Q3	Q4	Q5	Q6	Q7	Q8	Q9
Q2	0.272	‒							
Q3	0.183	0.321	‒						
Q4	0.301	0.488	0.475	‒					
Q5	0.256	0.186	0.316	0.267	‒				
Q6	0.055	0.359	0.395	0.431	0.172	‒			
Q7	0.159	0.289	0.229	0.401	0.216	0.241	‒		
Q8	0.281	0.282	0.593	0.489	0.374	0.272	0.235	‒	
Q9	0.255	0.467	0.210	0.326	0.210	0.263	0.413	0.237	‒
AKQoL-BR	0.450	0.654	0.614	0.781	0.475	0.563	0.583	0.630	0.643

The AKQoL-BR showed adequate temporal stability and sensitivity to improvement. The mean score (sd) of the tests and retests were 11.7 (6.5) and 11.3 (5.4), with an ICC of 0.88 (p < 0.01). The treated participants perceived a reduced impact on quality of life and decreased their scores from 7.0 (3.5) to 5.9 (3.5; p = 0.016).

Our results show that the AKQoL-BR proved to be feasible, consistent, reproducible, and sensitive for the assessment of AK impact on QOL in a Brazilian sample. The internal consistency was close to that of the original Danish version (α = 0.81), and of the other translations performed in Switzerland (α = 0.82) and Spain (α = 0.91); however, the Dutch version showed a less significant consistency (α = 0.64).^4^, ^5^, ^10^, ^11^ On the other hand, the analysis of the AKQoL-BR in subscales (function, emotions and control), conceived by the authors of the original scale, is not feasible in the Portuguese version, due to its unidimensionality.

Dermatological diseases affect different dimensions of life, due to symptoms, functional limitations and the promotion of stigmas associated with the appearance of lesions, which interfere with social interactions, professional activities, leisure and inflict damage on one’s self-esteem. Consequently, there may be psychological impairment of patients. The modern concept of medical treatment presupposes, in addition to the objective reduction of lesions, the effect of metrics based on patients' perceptions, such as the impact on QoL, which makes the use of specific instruments for QoL assessment in pre- and post-therapeutic evaluation relevant.

At the same time, it is important to emphasize that there is not always a perfect correlation between clinical severity and impact on QOL, as this is a subjective concept and depends on personal interpretations, which may vary between individuals, in different cultures, and even in the same individual, in different phases of one’s life. The meaning of the disease and its stigma is also highly variable, requiring care in the interpretation of outcomes reported by patients in heterogeneous populations. In this case, the floor effect observed in item 5, demonstrating a lack of concern with the camouflage of the lesions, might not be found in samples containing younger patients, or those who carry out social and work activities that require more frequent exposure of the lesions, as well as in patients with a higher educational level, who may have a different perception of the disease and, consequently, suffer a greater impact on quality of life. In the Dutch validation study, item 5 was also among those with a floor effect, in addition to items 3 and 8; while the Spanish study did not show any items with a floor effect.^5^

The present study has limitations, such as the predominance of elderly patients, attended in the public health system and with a low educational level, which makes it difficult to generalize the results; however, it does not undermine the properties of the validated instrument. The use of the AKQoL-BR in future studies will be important to consolidate its usefulness in assessing the quality of life of patients with AK.

In conclusion, a Brazilian Portuguese version of the AKQoL was adapted and validated, which showed favorable psychometric behavior for its use in clinical studies in patients with AK.

## Financial support

None declared.

## Authors' contributions

Marcella Akemi Haruno de Vilhena: Design and planning of the study; collection, analysis, and interpretation of data; Critical review of the literature, writing, and approval of the final version of the manuscript.

Ivanka Miranda de Castro: Collection, analysis, and interpretation of data; Critical review of the literature, writing and approval of the final version of the manuscript.

Anna Carolina Miola: Design and planning of the study; effective participation in research orientation; collection, analysis, and interpretation of data; critical review of the literature; critical review of the manuscript, writing and approval of the final version of the manuscript.

Ingrid Stresser Gioppo: Design and planning of the study; collection, analysis, and interpretation of data; critical review of the literature, approval of the final version of the manuscript.

Amanda Soares Teixeira: Design and planning of the study; analysis and interpretation of data; critical review of the literature, approval of the final version of the manuscript.

Hélio Amante Miot: Design and planning of the study; effective participation in research orientation, project development, analysis and interpretation of data; critical review of the literature; critical review of the manuscript, writing and approval of the final version of the manuscript.

## Conflicts of interest

None declared.
